# Once Forgotten Now Re-emerging: Scrub Typhus Infection in Pediatric Patients From North West India

**DOI:** 10.7759/cureus.44044

**Published:** 2023-08-24

**Authors:** Sujaya Mukhopadhyay, Rakesh Gupta, Shalini Shukla, Prasun Bhattacharjee, Ruchika Bhatnagar, Sanju Yadav, Sahabzada Faisal Kamal, Arashdeep Virk, Shazmeen Imran, Azhar Liyakath

**Affiliations:** 1 Pediatrics, Government Institute of Medical Sciences, Greater Noida, IND; 2 Pathology and Laboratory Medicine, Government Institute of Medical Sciences, Greater Noida, IND; 3 Pediatrics, Ananta Institute of Medical Sciences and Research, Rajsamand, IND; 4 Paediatrics, Government Institute of Medical Sciences, Greater Noida, IND

**Keywords:** pediatric scrub typhus, rajasthan, valvulitis, empyema, eschar, vasculitis, dengue mimic, acute undifferentiated fever, western uttar pradesh, mis-c mimic

## Abstract

Background

Scrub typhus is an important cause of acute febrile illness in children. It is one of the re-emerging infections in the Asia Pacific region. It is caused by the gram-negative bacteria *Orientia tsutsugamushi* and is spread by the bite of trombiculid mites. The initial symptomatology is nonspecific with fever, headache, vomiting, etc. The presence of eschar is said to be pathognomic. It is a systemic illness, and vasculitis is the basic pathogenic mechanism.

Materials and methods

A retrospective observational study was conducted in two medical colleges and associated hospitals of western Uttar Pradesh (UP) and Rajasthan, India. Case files of 21 confirmed cases of scrub typhus admitted from April 2021 to October 2022 were reviewed. Scrub typhus was suspected in children with acute undifferentiated fever, and suggestive signs and symptoms were confirmed serologically with IgM enzyme-linked immunoassay (ELISA). Demographic and clinical details were noted.

Results

During the study period, a total of 335 cases of acute undifferentiated fever were seen, and 6.2% of them were diagnosed as having scrub typhus infection on detailed investigation. The most common symptom was fever in 100% of them, vomiting in 57.1%, abdomen pain in 42.8%, and diarrhea in 19%. Maculopapular, erythematous rash was present in 19% of cases. None of the patients had eschar. Microvascular leakage was the main complication in 28.5%. Unusual complications seen were empyema and valvulitis in 4.7% of patients.

Conclusion

Scrub typhus is also seen in urban setups and in dry arid areas like Rajasthan and North West UP. So, relevant investigations should be a part of the evaluation in pediatric patients with acute undifferentiated fever. Eschar can be absent, and empyema and valvulitis are some uncommon complications. A high degree of suspicion and early diagnosis are essential as an undiagnosed infection is rapidly fatal.

## Introduction

Scrub typhus is an important cause of acute febrile illness in children in northern Australia, the western Pacific islands, and South Asia including India [1,2]. It is one of the re-emerging infections in the Asia Pacific region [3]. The causative agent of scrub typhus, the gram-negative bacteria *Orientia tsutsugamushi*, is spread by the bite of trombiculid mites that reside in moist soil [4]. Infected patients from scrub typhus and other rickettsial infections are usually not diagnosed in India due to their clinical presentation, which is nonspecific, and lack of knowledge and diagnostic facilities. A large number of studies across the world are based on adult populations. Most of the reports from India are from southern states or the Himalayan belt. Reports from the dry arid part of India like western Uttar Pradesh (UP) and Rajasthan states are scarce. Different studies from North India reported an incidence of 28% in the age group of 0-60 years [5], while different studies from Rajasthan reported an incidence of 23%-25% [6]. While data is available on infection in adults, there is a lack of data regarding the clinical profile of scrub typhus in the pediatric population from the northwest region of India [2,7].

After a bite of an infected chigger, the bacteria multiplies at the bite sites. Following an incubation period of five to 14 days, patients with scrub typhus develop nonspecific signs and symptoms like fever, rash, headache, myalgia, generalized lymphadenopathy, nausea, vomiting, and abdominal pain [3]. The clinical features vary from subclinical disease to organ failure and fatal disease. It is a systemic illness, and vasculitis is the basic pathogenic mechanism. There is endothelial cell involvement and perivascular infiltration of T cells and monocytes/macrophages. Inflammatory response involving several cytokines and causing tissue destruction follows. This immune response leads to various organ involvements and complications like hepatitis, meningoencephalitis, and acute respiratory distress syndrome (ARDS) [8]. Scrub typhus, if left untreated, can have fatal consequences.

To confirm scrub typhus infection, two types of laboratory investigations are available: (1) serological assays like the Weil-Felix test, indirect immunofluorescence assays, indirect immunoperoxidase assays, enzyme-linked immunosorbent assay (ELISA), and immunochromatographic tests (ICT) and (2) a molecular-based approach like polymerase chain reaction (PCR)-based tests.

PCR-based diagnostic tests have more specificity and sensitivity; however, genetic variations among strains of *O. tsutsugamushi* are the biggest hindrance to the diagnosis using the genetic marker via PCR. The indirect fluorescent antibody (IFA) test is considered a gold standard test for the detection of scrub typhus because of its higher sensitivity and specificity [9]. In comparison to the present gold standard method, IFA, enzyme‑linked immunosorbent assay (ELISA IgG and IgM) has comparable sensitivity and specificity, greater than 85%. So, it has become a better alternative in settings where the availability of costly equipment and trained personnel are limiting factors [10].

The recommended treatment regimen for scrub typhus is doxycycline (4 mg/kg/day PO or IV divided every 12 h; maximum: 200 mg/day). Alternative regimens include tetracycline (25-50 mg/kg/day PO divided every 6 h; maximum: 2 g/day) or chloramphenicol (50-100 mg/kg/day divided every 6 h IV; maximum: 4 g/24 h). Therapy should be continued for a minimum of five days and until the patient has been afebrile for at least three days to avoid relapse [11].

This study was undertaken to find out the incidence and clinic epidemiological profile of scrub typhus infection among the pediatric age group in northwest India.

## Materials and methods

The study was conducted after obtaining approval from the Institutional Ethical Committee of the Government Institute of Medical Sciences, Greater Noida, India (IEC No.: ECR/1224). It was a retrospective observational study, which was conducted in two medical colleges and associated hospitals of western Uttar Pradesh (UP) and Rajasthan, India. Case files of 21 patients between the age group of one month and 18 years with confirmed scrub typhus infection who were admitted from April 2021 to October 2022 in both institutes were reviewed, and data from the files were collected. Scrub Typhus Detect IgM ELISA Kit was used for serodiagnosis of scrub typhus infection. Demographic details, clinical features like fever duration, associated symptoms, presence of eschar, rash, organomegaly, laboratory data like complete blood counts (CBC), liver function tests (LFT), serum urea and creatinine, C-reactive protein (CRP), serum electrolytes, coagulation profile, ultrasound report, echocardiogram (ECHO), chest x-ray, investigations that were done to rule out differential diagnosis like rapid antigen test for malaria, Widal test for enteric fever, dengue IgM antibody and NS1 antigen test, and blood culture report were traced and noted.The presence of co-infection, data pertaining to the type of complications, need for oxygen support or mechanical ventilation, and final outcomes were also collected. The primary outcomes assessed were clinical and laboratory features, while the secondary outcomes were the final outcome and the presence or absence of co-infection.

Various definitions and the cutoffs used are listed in Table 1.

**Table 1:** Different definitions and cutoffs WBC: White blood cells; S. Na: Serum sodium; SGOT: Serum glutamic-oxaloacetic transaminase; SGPT: Serum glutamic pyruvic transaminase.

Various definitions	Cutoffs
Leukocytosis	WBC > 10,000 cells/mm^3^
Leukopenia	WBC < 5000 cells/mm^3^
Thrombocytopenia	Platelet count < 1,50,000/mm^3^
Hyponatremia	S. Na
SGOT elevation	SGOT > 40 U/L
SGPT elevation	SGPT > 40 U/L
Hypoalbuminemia	S. Albumin < 3.5 gm/dl

ARDS was defined as the acute onset of respiratory failure, bilateral infiltrates on chest radiograph, hypoxemia as defined by a PaO_2_/FiO_2_ ratio ≤ 200 mmHg, and no evidence of left atrial hypertension in the absence of heart failure [12].

Multiple organ dysfunction syndrome (MODS) is defined as the concurrent dysfunction of two or more organs or systems including respiratory, cardiovascular, hematological, neurological, gastrointestinal, hepatic, and renal [13].

The data was collected and analyzed by SPSS version 28 (IBM Corp., Armonk, NY). Descriptive statistics including frequency, mean, median, interquartile range (IQR), and standard deviation were calculated for the demographic data and laboratory parameters. Categorical variables were presented as percentages, and continuous variables were presented as mean/median along with a 95% confidence limit and IQR.

## Results

During the study period from April 2021 to October 2022, there were 185 cases of acute undifferentiated fever in the medical college from western UP and 150 in the medical college from Rajasthan. In total, 21 (6.2%; 21/335) cases were found to be positive for scrub typhus in the above-mentioned period: 11 (5.9%; 11/185) from western UP and 10 (6.6%; 10/150) from Rajasthan.

In the present study, 13 (61.9%) were males and eight (38.1%) were females. The majority of patients were above 10 years of age (Table 2).

**Table 2:** Demographic details AUF: Acute undifferentiated fever.

Characteristics	Total N (%) n=21
Male	13 (61.9)
Female	8 (38.1)
Number of cases from western UP	11
Number of AUF patients from western UP	185
% of patients with scrub typhus infection out of AUF patients from western UP (N = 185)	5.9%
Number of cases from Rajasthan	10
Number of AUF patients from Rajasthan	150
% of patients with scrub typhus infection out of AUF patients from Rajasthan (N = 150)	6.6%
Age	
<5 years	2 (9.5)
5-10 years	8 (38.1)
>10 years	11 (52.4)

The most common symptom noted was fever in 21 (100%), vomiting in 12 (57.1%), abdomen pain in nine (42.8%), and diarrhea in four (19%). Twelve (57.1%) patients had a fever for more than seven days, while one (4.7%) patient had a fever for two days only. Maculopapular, erythematous rash was present in four (19%) cases. None of the patients had eschar. Cough and difficulty breathing were seen in three (14.2%) patients. Three (14.2%) patients had hepatomegaly only, and three (14.2%) had isolated splenomegaly. Both hepatosplenomegaly was seen in four (19%) patients. Significant inguinal and mesenteric lymphadenopathy was seen in two (9.5%) patients. Dengue was the only co-infection seen. It was present in three (14.2%) patients (Table 3).

**Table 3:** Clinical features of the patients

Clinical feature	Total N (%), n = 21
Fever	21 (100)
Duration of fever	
<7 days	9 (42.9)
>7 days	12 (57.1)
Vomiting	12 (57.1)
Abdomen pain	9 (42.8)
Diarrhea	4 (19)
Headache	5 (23.8)
Eschar	0
Rash	4 (19)
Cough	3 (14.2)
Hepatomegaly	3 (14.2)
Splenomegaly	3 (14.2)
Hepatosplenomegaly	4 (19)
Significant lymphadenopathy	2 (9.5)
Dengue co-infection	3 (14.2)
Mortality	

In terms of complications seen, three (14.2%) had ARDS, and one (4.7%) had empyema, valvulitis, and shock. Meningoencephalitis was seen in two (9.5%) patients. Microvascular leakage was seen in six (28.5%) patients, ascites and edematous gallbladder (GB) wall were seen in four (19%) patients, while pleural effusion was seen in two (9.5%) patients. Bleeding was seen in two (9.5%) patients. Five (23.8%) patients needed oxygen support, and one (4.7%) patient was on ventilatory support (Table 4).

**Table 4:** Complications seen ARDS: Acute respiratory distress syndrome.

Complications	Total N (%), n = 21
Microvascular leakage	6 (28.5)
ARDS	3 (14.2)
Meningoencephalitis	2 (9.5)
Bleeding	2 (9.5)
Valvulitis	1 (4.7)
Empyema	1 (4.7)
Shock	1 (4.7)
Oxygen support	5 (23.8)
Mechanical ventilation	1 (4.7)

Laboratory investigations

In CBC, anemia was seen in 16 (76.1%) patients, leukocytosis in six (28.5%) patients, and leukopenia in five (23.8%) patients. Thrombocytopenia was seen in 12 (57.1%) patients. Serum albumin was low in 13 (61.9%) patients. Hyponatremia was seen in 14 (66.6%) patients and hypocalcemia in two (9.5%) patients. In one (4.7%) patient, PT/INR was prolonged, while activated partial thromboplastin time (APTT) was prolonged in two (9.5%) patients. SGOT and SGPT were deranged in 16 (76.1%) and 17 (80.9%) patients, respectively. CRP was above the cutoff in 10 (47.6%) cases. Blood culture was sterile in all the patients (Table 5).

**Table 5:** Lab abnormalities SGOT: Serum glutamic-oxaloacetic transaminase; SGPT: Serum glutamate pyruvate transaminase; CRP: C-reactive protein; PT/INR: Prothrombin time/International normalized ratio; APTT: Activated partial thromboplastin time.

Lab features	Total N (%), n = 21
Anemia	16 (76.1)
Leukocytosis	6 (28.5)
Leukopenia	5 (23.8)
Thrombocytopenia	12 (57.1)
Hyponatremia	14 (66.6)
Elevated SGOT	16 (76.1)
Elevated SGPT	17 (80.9)
Hypoalbuminemia	13 (61.9)
CRP raised (>5 mg/dl)	10 (47.6)
PT/INR prolongation	1 (4.7)
APTT prolongation	2 (9.5)
Hypocalcemia	2 (9.5)

Cerebrospinal fluid (CSF) examination was done in meningoencephalitis’ patients, which showed an increase in cell count (lymphocytic) and a mild increase in CSF protein.

All were treated as per protocol. There was no mortality. Some required high-grade antibiotics (vancomycin/linezolid) in view of septic shock and complicated pleural effusion features.

## Discussion

The distinctive feature of our study was the absence of eschar and the presence of empyema and valvulitis.

In this study, patients above 10 years of age were most frequently affected (52.4%), and infection was more common in boys than girls (1.6:1). In a study from Nepal [14], the female-to-male ratio was 1.15:1, and children between 10 and 14 years of age accounted for most of the cases (44%). This is similar in both the studies probably due to more outdoor activities leading to higher exposure to infected mites.

In the present study, fever was seen in 100%, vomiting in 57.1%, abdomen pain in 42.8%, and diarrhea in 19% of patients. Unlike reports from other studies [12], headache and myalgia were seen in only 23.8% of cases in our series. It was not a presenting complaint in our patients in contrast to the above studies. Similar to our cases, different studies have found fever and GI complaints to be the main symptoms [15,16]. Eschar was not seen in any patient in the present study. It is variably reported to be seen in 9%-46% of patients in India [17]. A study by Silpapojakul et al. [7] also did not report eschar in any of the reported cases. About 19% of patients in our study had erythematous eruptions involving palms and soles, which is again a very characteristic of this infection. Hepatosplenomegaly was seen in 47.6% of patients as was the case in another study from Nepal by Pathak et al. [18]. Empyema and valvulitis were the two unusual complications seen in our study, which are observed in 4.7% of patients each. The blood culture in the patient with empyema was negative. Empyema is not a very commonly reported complication in scrub typhus infection. The hypoalbuminemia leading to altered oncotic balance causes filtration of fluid in the pleural space. This along with microvascular injury in the lungs leads to noncardiogenic pulmonary edema causing effusion, and it can change to empyema in very few cases [19]. Agarwalla et al. from Orissa reported a case of a 20-month-old patient with scrub typhus infection and empyema [20]. Valvulitis is yet another cardiovascular complication that is reported rarely. Scrub typhus infection mainly causes conduction abnormalities, sometimes myocarditis and cardiomyopathy, and it leads to endocarditis rarely [21,22]. Valvulitis characterized by acute/subacute valvular regurgitation, cardiomegaly, and heart failure was seen in 4.7% of patients in our study. ECHO showed mild MR, TR, and AI with a normal ejection fraction of 65%. Creatine phosphokinase-MB (CPK-MB) and troponin T levels were within normal range. This was an unusual complication that we encountered. Similar to other studies from India [16], microvascular leakage in 28.5% was the most common complication seen in this study. Next was ARDS in 14.2%; meningoencephalitis and bleeding in 9.5%; and valvulitis, empyema, and shock in 4.7% each. Microvascular leakage occurs due to infection of the vascular endothelium [23]. It presents as pleural effusion, ascites, and edematous GB wall. ARDS pathogenesis is believed to be due to prolonged recruitment of inflammatory immune cells to the lung and damage to the vessels [24].

As in other studies [3,7,8,16], laboratory findings showed anemia in 76.1%, leukocytosis in 28.5%, leukopenia in 23.8%, thrombocytopenia in 57.1%, and hyponatremia in 66.6%. Deranged hepatic function in the form of elevated SGOT, SGPT, and hypoalbuminemia was found in 76.1%, 80.9%, and 61.9% of patients, respectively, in our study. CRP was raised in 47.6% of cases. Dengue was the only co-infection seen. It was present in 14.28% of patients.

The patients were managed as per protocol and responded to initial antibiotic therapy within two to three days. Everyone recovered and was discharged.

Strength

One of the strengths of the study was that it was conducted at centers where scrub typhus infection is not common and is usually not investigated. Some unusual complications of scrub typhus infection were detected among the patients. The study, though retrospective, generates some evidence that it is worth keeping scrub typhus infection in the differential diagnosis of AUF in these geographic regions.

Limitations

As this is a hospital-based study, it is difficult to assess the actual morbidity and mortality of the disease in these regions. Also, it is very difficult for younger children who are less than five years old to articulate a few symptoms. The small sample size and retrospective nature of the study are the other significant limitations.

## Conclusions

In any case of AUF in children, scrub typhus infection should be ruled out. It should be suspected even in urban and dry arid regions. Clinical features are varied and resemble a number of infectious diseases. The presence of eschar, though pathognomic, is not mandatory, and its absence should not be a criterion for not testing. There can be unusual complications like empyema and valvulitis. A high degree of suspicion and early diagnosis are essential as undiagnosed infection can lead to MODS with high case fatality rate.
